# A lightweight RPB-YOLO11-based detector improves mobile phenotyping of rice panicle blast

**DOI:** 10.3389/fpls.2026.1925522

**Published:** 2026-09-01

**Authors:** Xuzhe Yang, Xinbo Zhao, Chenming Xu, Juntao Hu, Liang Xu, Jianan Chi, Nannan Zhang, Xianlong Wang, Leilei Liu, Bing Liu, Liang Tang, Weixing Cao, Yan Zhu, Zaiwen Feng, Liujun Xiao

**Affiliations:** 1 National Engineering and Technology Center for Information Agriculture, Engineering Research Center of Smart Agriculture, Ministry of Education, Key Laboratory for Crop System Analysis and Decision Making, Ministry of Agriculture and Rural Affairs, Jiangsu Key Laboratory for Information Agriculture, Jiangsu Collaborative Innovation Center for Modern Crop Production, Nanjing Agricultural University, Nanjing, Jiangsu, China; 2 Key Laboratory of Tarim Oasis Agriculture (Tarim University), Ministry of Education, Alar, China; 3 School of Water Resources and Hydro-electric Engineering, Xi’an University of Technology, Xi’an, China; 4 College of Informatics, Huazhong Agricultural University, Wuhan, China

**Keywords:** rice panicle blast, plant disease detection, plant phenotyping, YOLO11, lightweight object detection, Android deployment

## Abstract

Rice panicle blast detection is an important task in plant disease phenotyping. Field-based detection remains challenging because infected spike regions are often small, sparse, elongated, and affected by overlapping panicles, complex backgrounds, and variable illumination. In this study, we propose RPB-YOLO11, a lightweight YOLO11-based detector designed for rice panicle blast detection. The model uses a Lightweight Ghost Backbone (LGB) to reduce redundant computation. It uses Anisotropic Axial Stripe Attention (A2SA) to represent elongated panicle structures. It also uses Focal Multi-Scale Attention (FMSA) for multi-scale feature refinement and Adaptive Geometric Shape IoU (AGS-IoU) for geometry-aware localization. The model was trained and evaluated on a rice panicle image dataset containing 1,055 training images, 69 validation images, and 169 test images. On the test set, RPB-YOLO11 achieved 76.09% mAP50, 45.44% mAP50-95, 73.98% precision, and 72.75% recall with 6.21 GFLOPs. Compared with the YOLO11n baseline, it improved mAP50, mAP50-95, precision, and recall by 2.73, 2.12, 1.64, and 2.11 percentage points, respectively. An Android-oriented inference application supports local image inference, detection visualization, class counting, and diseased-panicle incidence estimation. These results suggest that RPB-YOLO11 provides a practical approach for image-based rice panicle blast survey.

## 1 Introduction

Rice blast, caused by *Magnaporthe oryzae*, is one of the most destructive rice diseases and remains a persistent biological constraint on stable grain production (Wilson and Talbot, 2009; Skamnioti and Gurr, 2009; Asibi et al., 2019; Dean et al., 2012). Among its field manifestations, panicle blast is especially important for yield-oriented assessment because infection around the spike and neck can directly affect grain filling and harvestable output. Disease survey in paddy fields is still commonly supported by manual observation, yet visual scoring is laborious, subjective, and difficult to repeat consistently across dense plots, growth stages, and illumination conditions. Modern plant phenomics aims to convert sensor observations into quantitative biological and agronomic knowledge (Furbank and Tester, 2011; Araus and Cairns, 2014; Tardieu et al., 2017). In this setting, an image-based detector that localizes diseased panicle regions can provide spatially explicit evidence for disease monitoring instead of a single image-level label.

The need for reliable disease phenotyping has stimulated a broad transition from human visual estimates to sensor- and algorithm-assisted measurements. Imaging sensors can support more objective plant disease assessment, but disease symptoms vary with genotype, environment, organ position, imaging distance, and background composition (Mahlein, 2016; Bock et al., 2020). These factors are particularly severe in visible-range field images, where illumination changes, occlusion, symptom similarity, clutter, and unbalanced class occurrence remain major obstacles for automatic disease identification (Barbedo, 2016). For rice panicle blast, the practical bottleneck goes beyond recognizing whether disease is present. A useful field system must find small symptomatic spike regions, separate them from healthy panicles and leaves, and maintain enough speed and compactness for use close to the sensing device. Earlier work on rice panicle blast detection and grading supports the feasibility of panicle-level image assessment (Sethy et al., 2019). Most of this work relied on image processing descriptors or grading outputs rather than bounding box detection of individual panicles. Existing rice disease detectors also often focus on categories at the leaf level, which do not provide the diseased and total panicle counts needed for incidence estimation.

Deep learning has substantially advanced image-based plant disease recognition. Early convolutional classifiers showed that learned representations could identify plant diseases from leaf images under curated conditions (Mohanty et al., 2016; Sladojevic et al., 2016; Ferentinos, 2018). Subsequent surveys confirmed the rapid adoption of deep learning across agricultural vision tasks (Kamilaris and Prenafeta-Boldú, 2018). Object detection has extended disease recognition from image-level diagnosis to lesion or organ localization. This has been shown in rapid detection studies for crop diseases and pests (Fuentes et al., 2017). Mobile capture devices have also been explored for plant disease diagnosis, indicating the value of bringing inference closer to field users (Johannes et al., 2017; Deng et al., 2023). Recent rice-specific YOLO detectors have begun to address mobile, lightweight, and multi-scale disease detection scenarios (Deng et al., 2023; Trinh et al., 2024; Wang et al., 2025). Many plant disease systems are still evaluated on controlled or semi-controlled datasets or focus on leaf-level disease categories. In paddy-field panicle images, the detector must deal with repeated vegetation structures, overlapping organs, fine disease cues, and changing object scales.

General object detection research provides useful tools for this setting, including region-proposal detectors, feature pyramids, focal learning, and efficient detector scaling (Ren et al., 2017; Lin et al., 2017a, Lin et al., 2017b; Tan et al., 2020). One-stage detectors are attractive for agricultural deployment because they combine localization and classification in a single inference path. The YOLO family has become a common basis for fast object detection, from the original YOLO formulation to later efficient variants (Redmon et al., 2016; Bochkovskiy et al., 2020; Wang et al., 2023, Wang et al., 2024; Ultralytics, 2024). Direct adoption of a general YOLO detector does not fully resolve rice panicle blast phenotyping. Diseased spike regions may be small relative to the image, elongated along the panicle axis, and partially occluded by surrounding leaves. They can also appear visually close to healthy panicle tissue. At the same time, mobile or edge deployment discourages a larger backbone or heavy attention blocks.

Lightweight and edge-oriented model design provides a second line of prior work relevant to this problem. Compact convolutional networks such as MobileNet and GhostNet reduce redundant computation through depthwise or cheap feature-generation operations (Howard et al., 2017; Sandler et al., 2018; Han et al., 2020; Tang et al., 2022), while attention modules such as CBAM improve feature selectivity by recalibrating channel and spatial responses (Woo et al., 2018). In parallel, IoU-based bounding-box losses have improved localization learning by explicitly modeling overlap, center distance, and dynamic sample quality (Rezatofighi et al., 2019; Zheng et al., 2020; Tong et al., 2023). These studies suggest a route for rice panicle blast detection. They also leave an application-specific design gap. The detector should reduce redundant computation, model the anisotropic geometry of panicles, preserve multi-scale disease cues, and improve box regression without adding inference-time cost.

To address these gaps, we construct RPB-YOLO11, a lightweight improved YOLO11 detector for rice panicle blast detection and Android-oriented edge deployment. Each modification targets a failure mode expected in field panicle images. The Lightweight Ghost Backbone (LGB) uses GhostNetV2-style feature generation to reduce redundant backbone computation while retaining high-level context. Anisotropic Axial Stripe Attention (A2SA) is placed after SPPF to model elongated panicle structures through stripe convolution, scale gating, and axial attention. Focal Multi-Scale Attention (FMSA) refines the P4 neck branch so that small diseased spikes and larger panicle tissues remain represented under scale variation. Adaptive Geometric Shape IoU (AGS-IoU) changes only the training-time localization loss by adding direction-, shape-, and quality-aware box supervision. The resulting detector is evaluated for small panicleblast localization, cluttered paddy backgrounds, compact computation, and local image inference on Android.

## 2 Materials and methods

### 2.1 Dataset and training setup

**Figure 1:**
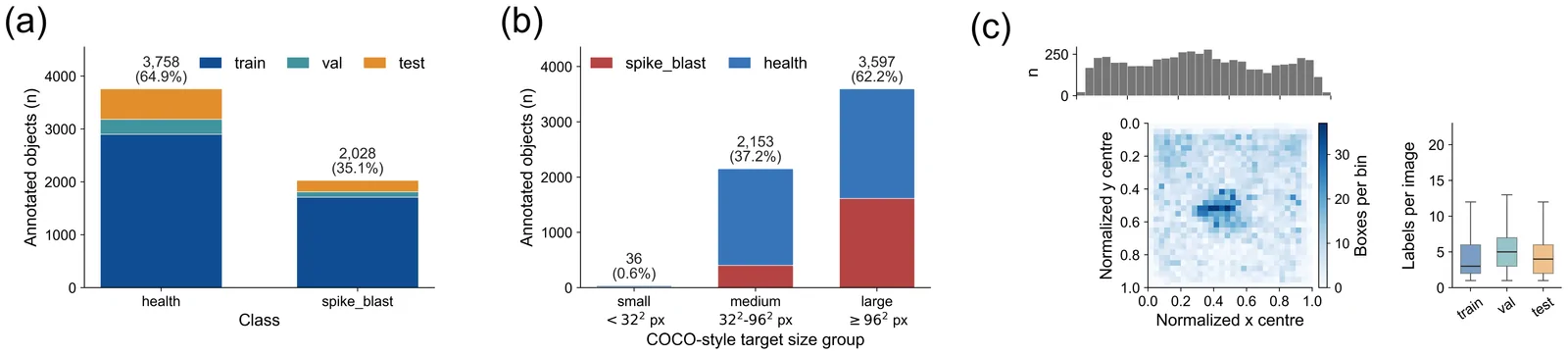
Dataset annotation characteristics used for rice panicle blast detection. **(a)** Class-wise annotatedobject counts across training, validation, and test splits. **(b)** COCO-style target-size distribution for spike_blast and health instances. **(c)** Spatial distribution of annotation centers and label counts per image.

For image-based phenotyping of rice panicle blast under field conditions, a detection dataset was established with separate training, validation, and test subsets (https://huggingface.co/datasets/anthony2261/paddy-disease-classification). The original dataset was randomly divided at an 8:1:1 ratio. The training subset was then augmented to expand the images used for model fitting, while the validation and initial test subsets were kept independent from training. To stabilize final evaluation, 100 independent original field images with paired labels were added only to the test subset. After augmenting the training set and supplementing the test set, the final dataset comprised 1,055 training images, 69 validation images, and 169 test images. These final subsets corresponded to 81.6%, 5.3%, and 13.1% of the 1,293 images, respectively. The training set contained 4,610 annotated objects, including 1,710 spike_blast and 2,900 health instances. The validation set contained 388 annotated objects, including 104 spike_blast and 284 health instances. The test set contained 788 annotated objects, including 214 spike_blast and 574 health instances. **Figure 1** summarizes the class composition, target-size distribution, spatial distribution of annotations, and the number of labels per image.

All models were trained with an input size of 640 pixels, batch size of 64, and a maximum schedule of 200 epochs. Early stopping was enabled, so curves for early-stopped runs end at their last completed epoch. The experiments were conducted on a workstation with an NVIDIA RTX 4090 GPU with 24 GB memory. The workstation also used an Intel Xeon Gold 5418Y CPU with 10 cores and 120 GB system memory. The software environment used Python 3.10 and PyTorch 2.2.2. The training configuration used stochastic gradient descent, an initial learning rate of 0.01, weight decay of 0.0005, mosaic augmentation, HSV color augmentation, horizontal flipping, and close-mosaic scheduling. A warmup stage of 3 epochs was used, with warmup momentum of 0.8 and warmup bias learning rate of 0.1. The same batch size, warmup schedule, and augmentation settings were kept across all YOLO models. This design made the baseline and improved YOLO models comparable under the same training protocol. RT-DETRv2-S was evaluated on the same data split, and its curves were plotted from epoch-level validation metrics.

### 2.2 Model construction

**Figure 2:**
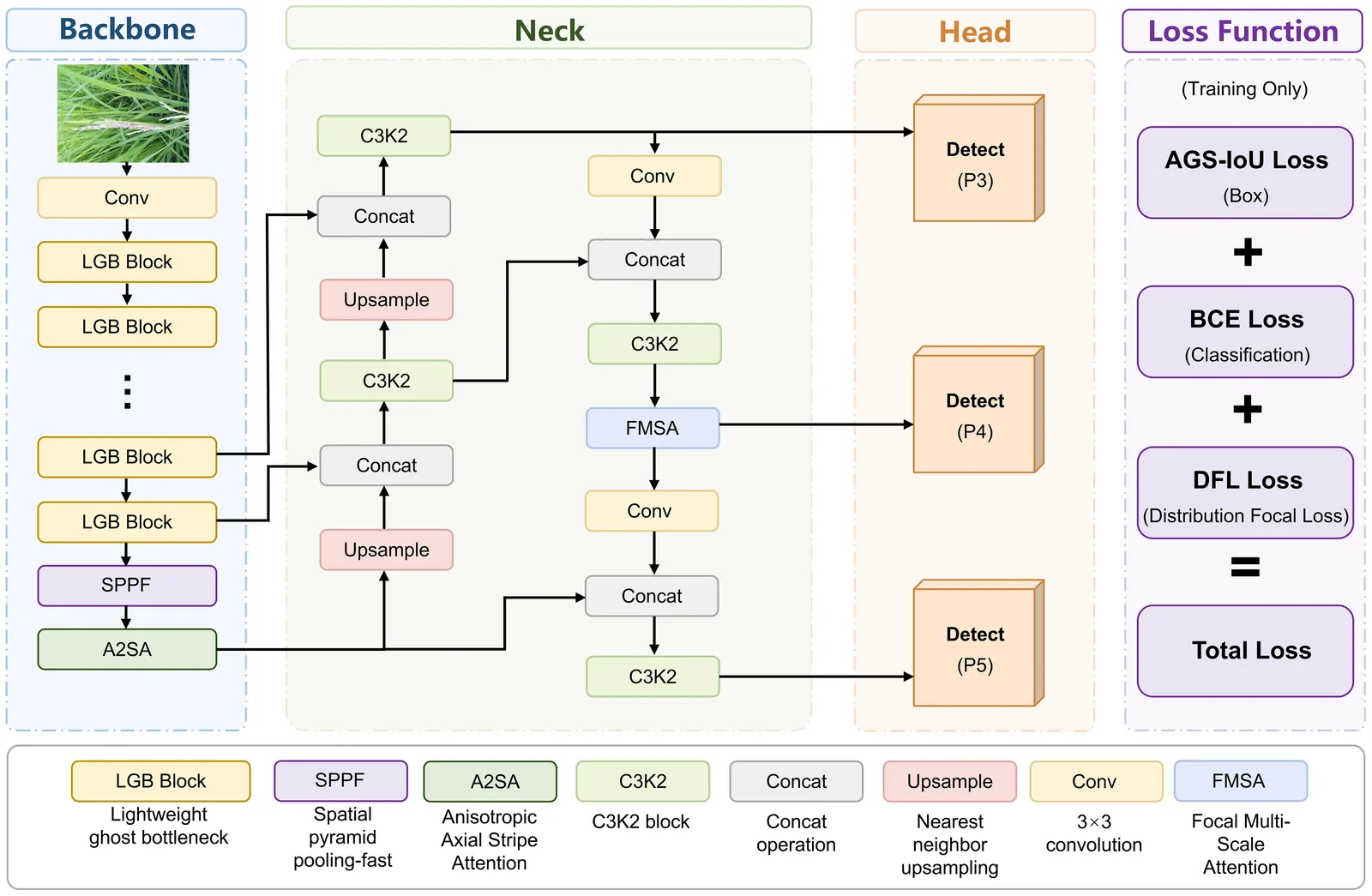
Overall architecture of RPB-YOLO11 for rice panicle blast detection.

RPB-YOLO11 was constructed as a lightweight one-stage detector for localizing rice panicle blast and healthy panicle instances in field images. The design retained the three-scale detection principle of YOLO11, in which feature maps at P3, P4, and P5 are used to detect objects at different spatial resolutions (Ultralytics, 2024). The feature extractor, high-level attention stage, neck refinement, and localization loss were modified to better match the visual structure of panicles and the computational constraints of mobile edge phenotyping. The overall architecture is shown in **Figure 2**. The detector used the two-class output definition described in Section 2.1.

The backbone was redesigned to reduce redundant convolutional computation while preserving multiresolution representations. The original YOLO11n backbone is based on repeated convolution and C3K2 stages, followed by SPPF and C2PSA. In RPB-YOLO11, these early and middle feature-extraction stages were replaced by a Lightweight Ghost Backbone (LGB) built from GhostNetV2-style feature generation (Han et al., 2020; Tang et al., 2022). Ghost modules first generate intrinsic feature maps with a small number of standard convolutions and then expand them through cheaper linear or depthwise operations. This choice was made because repeated leaf and panicle textures can produce redundant feature responses in paddy-field images. The GhostNetV2-style backbone further introduces decoupled fully connected spatial encoding, which improves long-range spatial representation without replacing the detector with a heavy transformer-like backbone. The backbone output was passed through SPPF to aggregate multi-scale context before attention refinement.

A2SA was inserted after SPPF to strengthen high-level directional representation for elongated panicle structures. The module follows a split-merge pattern similar to the C2 family of YOLO blocks. Given an input feature map, A2SA first applies parallel depthwise strip convolutions with fine and coarse receptive fields. It uses strip kernels of 7 and 13 pixels along vertical and horizontal directions, followed by a scale gate that fuses fine-scale and coarse-scale responses from global context. The fused feature is then processed by anisotropic axial attention, where two-dimensional attention is decomposed into sequential vertical and horizontal passes. This decomposition is intended to model the main axis of panicles and their local separation from neighboring vegetation while avoiding the full cost of global two-dimensional self-attention. In the proposed detector, A2SA replaces the original C2PSA stage at the high-level backbone output.

The neck follows a feature-pyramid and path-aggregation logic for multi-scale detection (Lin et al., 2017a). High-level features are upsampled and concatenated with lower-level backbone features to form the P3 branch, and the fused representations are subsequently downsampled and aggregated for P4 and P5 detection. Diseased spike regions can appear as small local symptoms. Healthy panicles and large diseased regions may occupy larger image areas. To improve the medium-scale branch, FMSA was inserted after the P4 C3K2 block. FMSA applies depthwise convolutional banks with 3, 5, and 7 pixel kernels. It then computes spatial energy across scales, reweights scale responses with a focal modulation term, and fuses them with learnable per-channel scale weights. A lightweight squeeze-and-excitation recalibration is applied after fusion. The residual coefficient is initialized at zero, so the module begins close to an identity mapping and can learn scale-sensitive refinement progressively (Woo et al., 2018; Lin et al., 2017b).

The detection head predicts bounding boxes and class probabilities from P3, P4, and P5 feature maps. Compared with the baseline model, the head topology was kept largely unchanged so that the contribution of LGB, A2SA, FMSA, and the modified localization loss could be isolated in later ablation experiments. During training, the default box-regression objective was replaced with AGS-IoU. AGS-IoU computes the localization score as an IoU term penalized by an adaptive geometric term that combines directionmodulated center distance and shape-aware width-height discrepancy. The adaptive scale factor is updated from the running quality of predictions and is constrained to a valid numerical range, which was intended to reduce unstable gradients during box optimization. The total training objective combines the AGS-IoU box term, BCE classification loss, and distribution focal loss (DFL). Because AGS-IoU is used only in loss computation, it changes the learned detector but does not add operators to the inference graph, which is important for subsequent Android-oriented deployment (Rezatofighi et al., 2019; Zheng et al., 2020; Tong et al., 2023).

LGB and A2SA operate in the backbone and are described in Section 2.3. FMSA operates in the neck and refines the P4 branch, as detailed in Section 2.4. AGS-IoU operates only during optimization, as described in Section 2.5. This separation keeps the inference pathway compact while allowing the training objective to place stronger geometric constraints on predicted bounding boxes.

### 2.3 LGB and A2SA

LGB and A2SA were placed in the backbone because rice panicle blast detection depends strongly on early-to-high-level visual representation. The two modules have complementary roles. LGB reduces redundant convolutional feature generation while preserving the staged pyramid structure required by the detection neck. A2SA then refines the high-level backbone output by modeling the anisotropic geometry of panicles and the scale variation of disease regions. Unless otherwise stated, $X\in {\mathbb{R}}^{C\times H\times W}$ denotes a feature map with *C* channels and spatial size *H* × *W*. The symbols ʘ, Concat(·), *σ*(·), *δ*(·), GAP(·), FFN(·), $D_{a\times b}\left(\cdot \right)$, and $C_{a\times b}\left(\cdot \right)$ denote element-wise multiplication, channel-wise concatenation, the sigmoid function, the ReLU activation, global average pooling, a pointwise feed-forward transformation, depthwise convolution, and standard convolution, respectively.

#### 2.3.1 Lightweight ghost backbone

**Figure 3:**
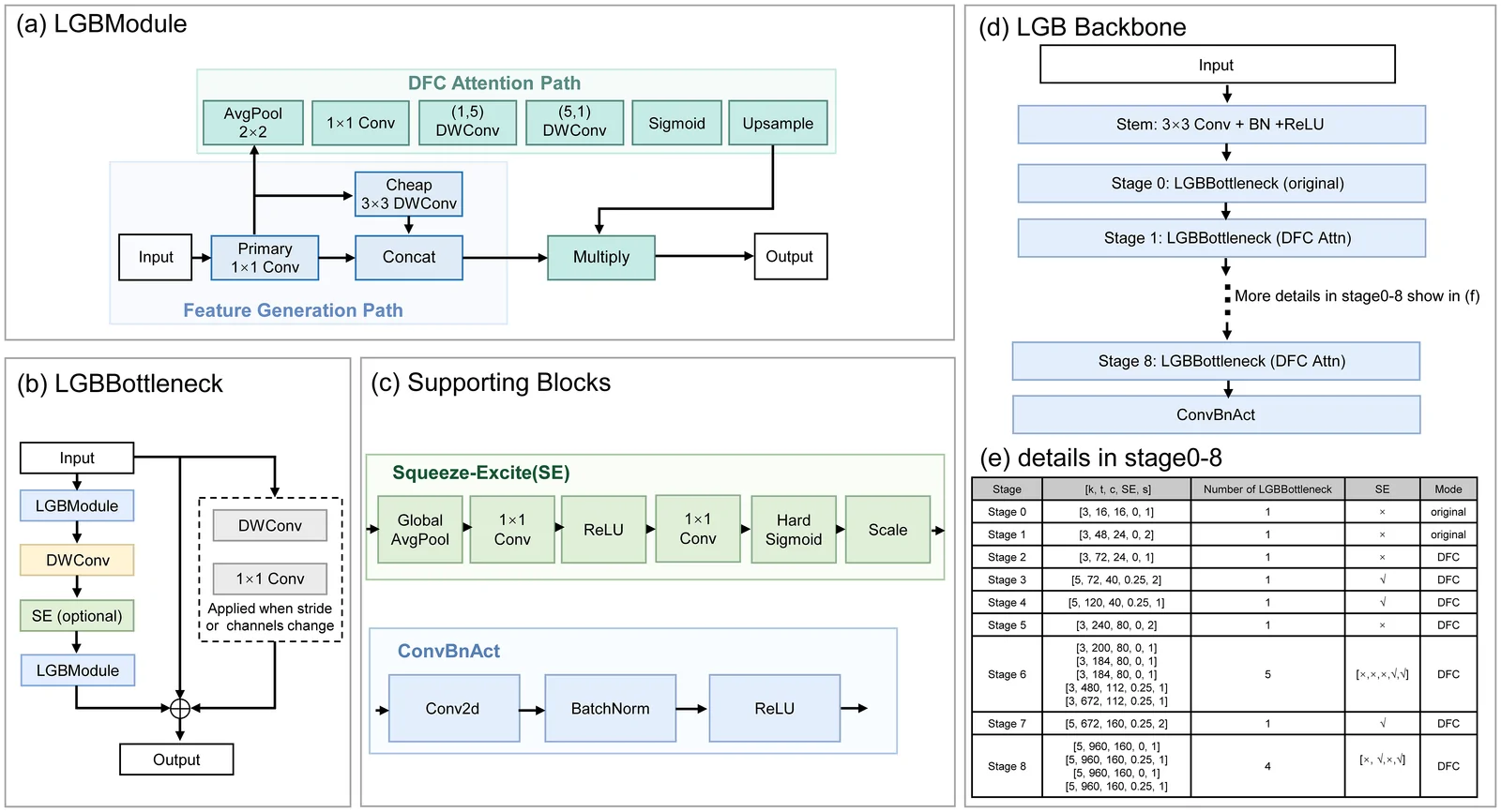
Lightweight Ghost Backbone (LGB) used in RPB-YOLO11. **(a)** LGB module with a Ghost feature generation path and DFC attention path. **(b)** LGB bottleneck with depthwise convolution, optional SE recalibration, and shortcut connection. **(c)** Supporting SE and convolution-normalization-activation blocks. **(d)** Stage-level LGB backbone organization and stage settings. **(e)** details in LGB backbone.

The LGB module was used to replace repeated standard convolutional feature extraction with GhostNetV2style feature generation (Han et al., 2020; Tang et al., 2022). The motivation is that visible paddy-field images contain many repeated leaf and panicle textures. In such scenes, producing all output channels with independent standard convolutions can be inefficient. Ghost feature generation first computes a compact intrinsic representation and then derives additional feature maps through cheaper transformations. As shown in **Figure 3**, LGB consists of a feature generation path, a decoupled fully connected (DFC) attention path, optional squeeze-and-excitation (SE) recalibration, and residual bottleneck connections.

Given an input feature map *X*, the LGB feature generation path first computes an intrinsic feature map *X_p_* with a primary convolution and then generates additional ghost features *X_g_* with a cheap depthwise operation. The output feature map *Y_g_* is obtained by concatenating and truncating these features to the required output channel number *C_o_* Equation 1:

(1) $$X_{p}=C_{1\times 1}\left(X\right),\;X_{ɡ}=D_{3\times 3}\left(X_{p}\right),\;Y_{ɡ}=\text{Concat }{\left(X_{p},X_{ɡ}\right)}_{1:C_{o}}.$$

Here, *X_p_* represents intrinsic features, *X_g_* represents inexpensive ghost features, and the subscript 1: *C_o_* denotes channel selection when the concatenated feature map contains more channels than required. This operation follows the principle that many convolutional responses can be approximated from a smaller set of intrinsic maps (Han et al., 2020).

To compensate for the limited spatial context of lightweight operations, LGB introduces a DFC attention gate in deeper stages. The gate reduces the spatial resolution by average pooling, applies a pointwise convolution, and then uses 1×5 and 5×1 depthwise convolutions to encode horizontal and vertical context. The resulting sigmoid attention map is upsampled to the size of *Y_g_*and multiplied with the ghost feature map. Each LGB bottleneck also includes optional spatial downsampling, optional SE recalibration, and a residual shortcut. In the SE branch, an intermediate feature *Z* is compressed by global average pooling, mixed by two channel transformations *W*_1_ and *W*_2_, and used to reweight *Z*. This recalibration lets the backbone emphasize informative channels while retaining a compact convolutional structure (Hu et al., 2018). In the present detector, LGB provides multi-stage feature maps for subsequent SPPF and A2SA processing. This supports compact feature extraction before the neck performs multi-scale fusion.

#### 2.3.2 Anisotropic axial stripe attention

**Figure 4:**
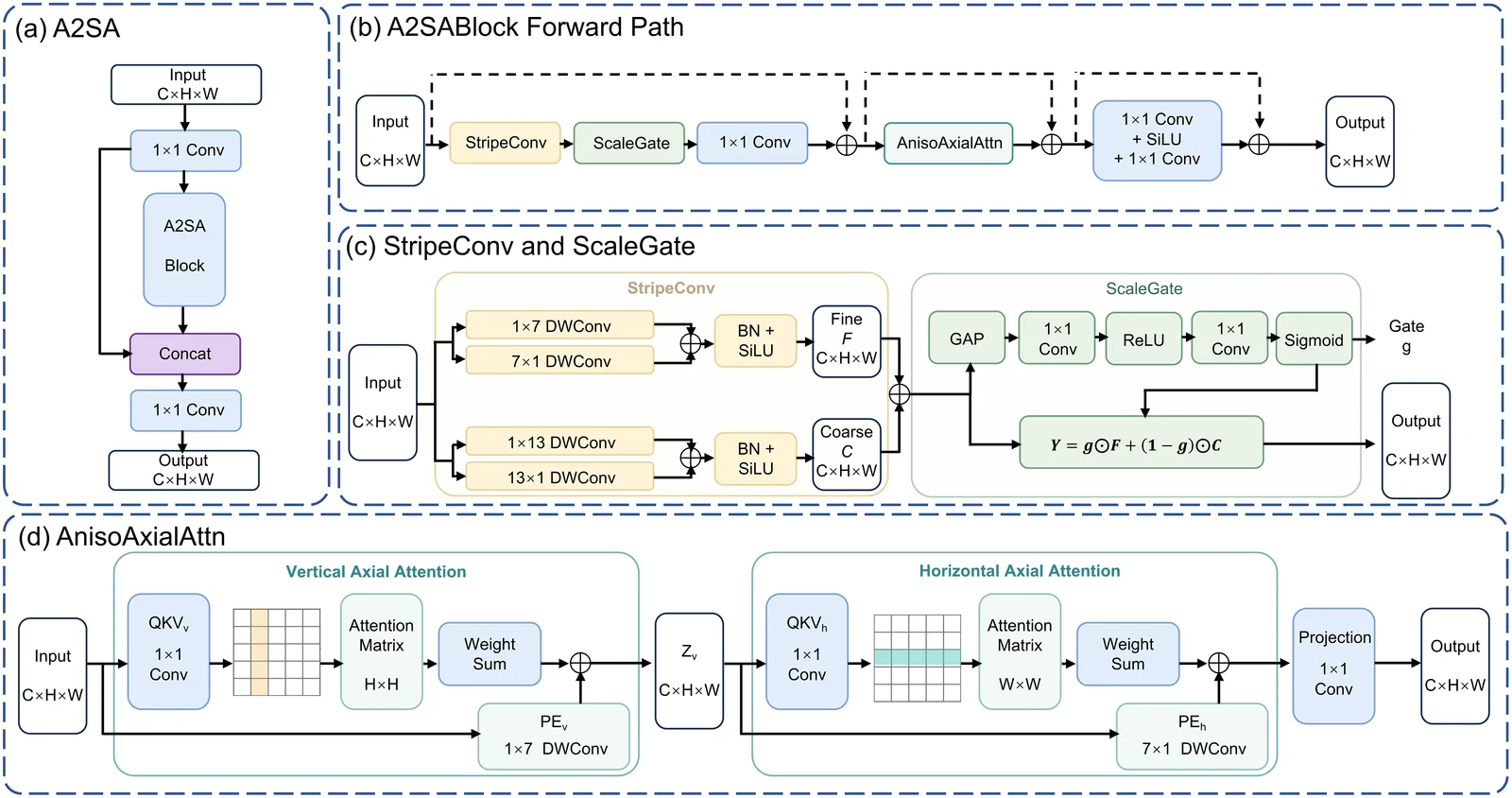
Anisotropic axial stripe attention (A2SA) for high-level backbone refinement. **(a)** Split-merge container of A2SA. **(b)** Forward path of the A2SA block with residual connections. **(c)** StripeConv and ScaleGate, where fine and coarse strip-convolution responses are adaptively fused. **(d)** Anisotropic axial attention, where vertical and horizontal attention are computed sequentially with strip-shaped positional encoding.

A2SA was added after SPPF to refine the high-level backbone feature map for elongated rice panicle structures. Panicles and diseased spike regions often form narrow, directionally biased targets, while surrounding leaves and neighboring panicles create dense background interference. A standard twodimensional attention operation can model global context but has quadratic complexity over all spatial tokens (Vaswani et al., 2017). A2SA reduces this cost by using strip-shaped convolution for directional local cues, scale-adaptive gating for fine and coarse stripe responses, and axial attention for sequential vertical and horizontal context modeling (**Figure 4**).

A2SA first uses a split-merge container to preserve a direct information path while refining only part of the feature representation. Given *X*, a 1 × 1 projection produces two channel groups *X_a_*and *X_b_*. The second group is processed by *n* stacked A2SA blocks. The two groups are then concatenated and projected Equation 2:

(2) $$\left[X_{a},X_{b}\right]=\text{Split}\left(C_{1\times 1}\left(X\right)\right),Y_{\text{A}2\text{SA}}=C_{1\times 1}\left[\text{Concat}\left(X_{a},A^{\left(n\right)}\left(X_{b}\right)\right)\right],$$

where Split(·) divides channels into two equal groups, and $A^{\left(n\right)}\left(\cdot \right)$ denotes *n* repeated A2SA blocks. One channel group keeps a direct shortcut, and the other group is used for direction- and scale-aware refinement.

Inside each A2SA block, strip convolution extracts fine and coarse directional features. Fine-scale features *F* are computed with 1 × 7 and 7 × 1 depthwise convolutions. Coarse-scale features *R* are computed with 1 × 13 and 13 × 1 depthwise convolutions. A per-channel scale gate *g* ∈ [0,1]*^C^*, estimated by a pooled two-layer channel mapping *g* = *σ*(*W*_2_*δ*(*W*_1_ GAP(*F* + *R*))), fuses the two responses as *S* = *g* ʘ *F* + (1 − *g*) ʘ *R*. Channels that respond to fine disease boundaries can emphasize *F*. Channels requiring broader panicle context can emphasize *R*. This channel-wise scale selection is consistent with attention-based feature recalibration (Hu et al., 2018; Woo et al., 2018).

After stripe fusion, A2SA applies vertical and horizontal axial attention in sequence, denoted as $\text{AxialAttn}(U)={\text{Attn}}_{W}({\text{Attn}}_{H}(U))$. Vertical attention treats each column as a sequence of length *H*, and horizontal attention treats each row as a sequence√ of length *W*. In each axial pass, query, key, and value projections are used to compute $\text{softmax}(QK^{⊤}/\sqrt{d})V_{\text{val}}$, where *d* is the key dimension in each head and *V*_val_ denotes the value projection. Strip-shaped positional encodings are added to retain directional location information. The A2SA block output aggregates stripe fusion, axial attention, and a feed-forward transformation through residual updates Equation 3:

(3) $$U=X+C_{1\times 1}\left(S\right),\;Z=U+\text{AxialAttn}(U),O=Z+\text{FFN}(Z),$$

where *U* is the stripe-enhanced feature, *Z* is the feature after axial attention, and *O* is the output of one A2SA block. Compared with full spatial self-attention over all *HW* tokens, the axial decomposition reduces the dominant spatial complexity from $O\left(H^{2}W^{2}d\right)$ to $O\left(\left(H^{2}W+HW^{2}\right)d\right)$. This reduction follows the same rationale as axial attention for dense prediction (Wang et al., 2020), but here it is adapted to the directional morphology of rice panicles. Because A2SA is applied to low-resolution high-level feature maps, its main role is direction-aware context modeling rather than a large absolute reduction in whole-model computation.

### 2.4 Focal multi-scale attention

**Figure 5:**
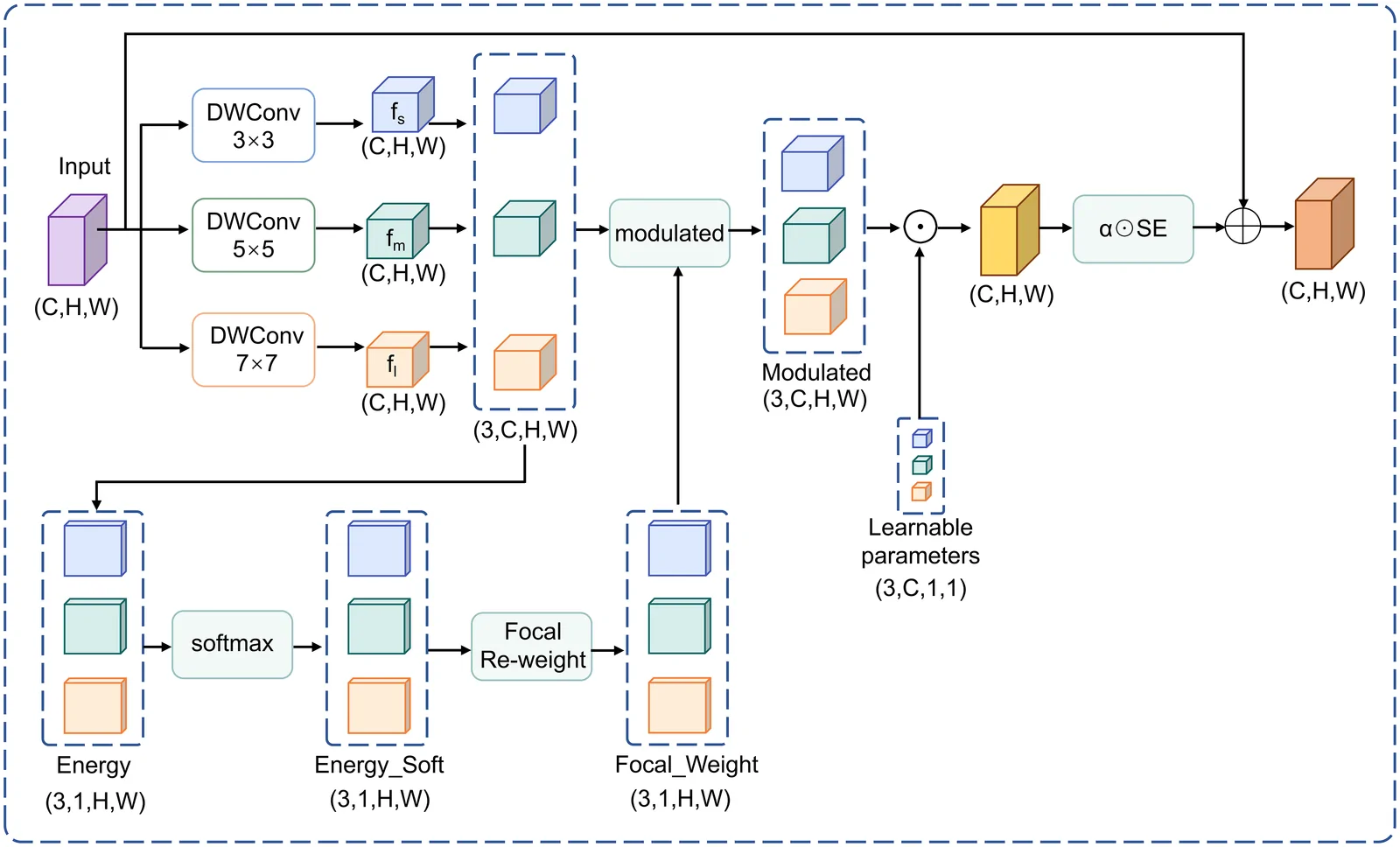
Focal multi-scale attention (FMSA) used to refine the P4 branch in RPB-YOLO11. The input feature map is processed by parallel 3, 5, and 7 pixel depthwise convolutions to extract fine-, medium-, and coarse-scale responses. Spatial energy is computed for each scale, normalized across scales, and transformed into focal scale weights. The modulated features are fused with learnable per-channel scale parameters, recalibrated by SE, and injected through a residual path controlled by a learnable coefficient.

Focal Multi-Scale Attention (FMSA) was introduced into the neck to refine the P4 feature branch before medium-scale detection. The neck already aggregates semantic and spatial information across pyramid levels, following the general rationale of feature-pyramid detection (Lin et al., 2017a). Rice panicle blast images contain a mixture of small diseased spike regions, medium panicle parts, and larger healthy panicle structures. If all spatial scales are treated uniformly, features associated with frequent or visually dominant object sizes can suppress weaker disease-related responses. FMSA was designed to counter this scale competition in three steps: extracting depthwise responses at 3, 5, and 7 pixel kernels, normalizing spatial energy across these responses, and injecting the recalibrated feature through a residual path (**Figure 5**). The design is related to prior work on depthwise separable convolution, attention recalibration, and focal weighting, but adapts these ideas to scale competition in the detection neck (Chollet, 2017; Hu et al., 2018; Woo et al., 2018; Lin et al., 2017b).

The notation follows Section 2.3. Let $X\in {\mathbb{R}}^{C\times H\times W}$ denote the P4 feature map entering FMSA, and let K={3,5,7} be the set of convolutional kernel sizes. For each scale k∈K, FMSA computes a depthwise convolutional response Equation 4.

(4) $$F_{k}=D_{k\times k}\left(X\right),\;k\in \left\{3,5,7\right\},$$

where *F*_3_, *F*_5_, and *F*_7_ correspond to fine-, medium-, and coarse-scale receptive fields, respectively. The use of depthwise convolution keeps the operation channel-wise and computationally compact, which is important because FMSA is inserted into the inference path rather than used only during training (Chollet, 2017; Sandler et al., 2018).

To measure which scale dominates each spatial location, FMSA averages the squared response of *F_k_*over channels and normalizes the resulting energy maps across the three scales. Specifically, $e_{k}\left(h,w\right)=C^{-1}\sum_{c}F_{k}{\left(c,h,w\right)}^{2}$, and $p_{k}\left(h,w\right)$ is obtained by applying a softmax over k∈{3,5,7} at each location. The normalized energy value $p_{k}\left(h,w\right)$ describes the relative dominance of scale *k* at location (*h,w*). A focal reweighting term ${\left(1-p_{k}\right)}^{\gamma }$, with *γ* = 8 in the implemented model configuration, suppresses locally dominant scales and increases the relative contribution of weaker scales. These focal weights are normalized across scales before being multiplied with the scale responses. This step encourages the neck to retain information from less dominant receptive fields, which is useful when small blast symptoms coexist with larger panicle structures (Lin et al., 2017b).

After focal modulation, FMSA performs learnable channel-wise scale fusion. Let λ*_k_*,*_c_* denote the normalized learnable coefficient for scale *k* and channel *c*, and let ${\tilde{F}}_{k}$ denote the focal-modulated version of *F_k_*. The fused feature *M* is formed as $M\left(c,h,w\right)=\sum_{k\in K}\lambda_{k,c}{\tilde{F}}_{k}\left(c,h,w\right)$. This formulation lets each channel learn its preferred receptive field rather than forcing the same scale mixture for all semantic responses. Such adaptive scale selection is conceptually related to selective-kernel and attention-based recalibration mechanisms (Li et al., 2019; Hu et al., 2018; Woo et al., 2018).

Finally, the fused feature is recalibrated by an SE-style channel gate and added back to the original feature through a learnable residual coefficient Equation 5:

(5) $$Y=X+\alpha \left[Mʘ\sigma \left(W_{2}\delta \left(W_{1}\text{GAP}(M)\right)\right)\right],$$

where *Y* is the FMSA output, *W*_1_ and *W*_2_ are channel-mixing transformations, and *α* is a learnable residual coefficient. The coefficient *α* is initialized so that the module starts close to an identity mapping. This residual form limits disruption of the original neck representation at the start of training while allowing scale-aware feature enhancement to be learned progressively. In RPB-YOLO11, the FMSA output is passed to the P4 detection branch and to the subsequent downsampling path, so the refined medium-scale representation can also influence the higher-level P5 aggregation.

### 2.5 AGS-IoU loss

The bounding-box regression objective was redesigned to improve localization learning for sparse and elongated rice panicle blast regions. Classical IoU loss aligns the optimization target with the detection metric, but it provides weak guidance when boxes do not overlap. GIoU, DIoU, and CIoU address this limitation by adding enclosing-box, center-distance, and aspect-ratio constraints. WIoU further introduces a dynamic focusing mechanism to adjust the contribution of samples with different localization quality (Rezatofighi et al., 2019; Zheng et al., 2020; Tong et al., 2023). For panicle blast detection, localization errors are often directional and shape dependent. A small shift along the panicle axis and a similar shift across adjacent panicles can have different effects on the final annotation match. Adaptive Geometric Shape IoU (AGS-IoU) defines the regression score from overlap, a direction-aware center penalty, a shape-aware width-height penalty, and an adaptive quality scale. The loss is used only during training and does not alter the inference graph.

For the *i*-th foreground prediction, let $B_{i}=\left(x_{i},y_{i},w_{i},h_{i}\right)$ denote the predicted box in center-size form and $B_{i}^{*}=\left(x_{i}^{*},y_{i}^{*},w_{i}^{*},h_{i}^{*}\right)$ denote the matched ground-truth box. The standard overlap term is Equation 6.

(6) $$I_{i}=\frac{\left|B_{i}\cap B_{i}^{*}\right|}{\left|B_{i}\cup B_{i}^{*}\right|+\epsilon },$$

where *I_i_* is the IoU value, |·| denotes area, and *ϵ* is a small constant for numerical stability. Let *c_w_*,*_i_* and *c_h_*,*_i_* be the width and height of the smallest enclosing box covering *B_i_* and $B_{i}^{*}$, and define the center offsets as $\Delta x_{i}=x_{i}^{*}-x_{i}$ and $\Delta y_{i}=y_{i}^{*}-y_{i}$. Inspired by direction-aware box regression in SIoU (Gevorgyan, 2022), AGS-IoU replaces angle-chain calculations with a smooth horizontal-orientation factor. The horizontal-orientation factor is computed from the relative contribution of $\left|\Delta x_{i}\right|$ to the center displacement. In the implementation, $q_{i}=\left|\Delta x_{i}\right|/\left(\sqrt{\text{Δ}x_{i}^{2}+\text{Δ}y_{i}^{2}}+\epsilon \right)$ is mapped by a smooth logistic function $a_{i}={\left[1+\exp\left(-5\left(q_{i}-0.5\right)\right)\right]}^{-1}$. The direction-modulated penalty is then $R_{\text{dir},i}=a_{i}{\left(\text{Δ}x_{i}/\left(c_{w,i}+\epsilon \right)\right)}^{2}+\left(1-a_{i}\right){\left(\text{Δ}y_{i}/\left(c_{h,i}+\epsilon \right)\right)}^{2}$. Compared with an isotropic center-distance penalty, *R*_dir,_*_i_* lets the regression objective adapt to whether the dominant mismatch is horizontal, vertical, or diagonal. This is useful for rice panicle scenes because neighboring panicles and leaves often create elongated, directionally biased localization ambiguity.

AGS-IoU also includes a shape-aware term *R*_shape,_*_i_* to penalize width and height mismatch. Following the principle that box shape and scale can influence regression behavior (Zhang and Zhang, 2023), the implementation retains the Shape-IoU-style normalized width and height discrepancies but uses shape exponent *s* = 0, giving balanced width and height coefficients. The relative discrepancies *ω_w,i_* and *ω_h,i_* are normalized by the larger predicted or target side length. The penalty is $R_{\text{shape,}i}=\left(1-\exp\left(-3\omega_{w,i}\right)\right)+\left(1-\exp\left(-3\omega_{h,i}\right)\right)$, so it remains differentiable while penalizing aspect and side-length mismatch in panicle-related boxes.

To avoid assigning the same geometric penalty to all foreground samples, AGS-IoU uses an adaptive scale factor *S_i_* derived from the running localization gap. Let $g_{i}=\text{clamp}\left(1-I_{i},0,1\right)$ denote the IoU gap and let ℱ be the set of foreground predictions in a mini-batch. The running mean gap ${\bar{g}}_{t}$ is updated by exponential moving average with update rate $m_{s}=1-0.5^{1/7000}$ and a lower bound of 10^−7^. The relative gap $\beta_{i}=g_{i}/{\bar{g}}_{t}$ is clamped to [0,15], and the implemented non-monotonic focusing curve uses $\alpha_{i}=\delta_{s}\gamma_{s}^{\beta_{i}-\delta_{s}}$ and $S_{i}=\text{clamp}\left(\beta_{i}/\alpha_{i},0,1\right)$, with $\gamma_{s}=1.5$ and $\delta_{s}=2.5$. In this setting, ordinary-quality predictions receive stronger regression emphasis, while high-quality boxes and extreme outliers are prevented from dominating the gradient allocation (Tong et al., 2023).

The AGS-IoU score is defined by subtracting the scaled geometric penalty from the overlap term Equation 7:

(7) $$I_{\text{AGS},i}=I_{i}-S_{i}\left(R_{\text{dir},i}+0.5R_{\text{shape},i}\right).$$

where *I*_AGS_*_,i_* is the AGS-IoU score, *R*_dir_*_,i_*is the direction-modulated distance penalty, *R*_shape_*_,i_* is the shape-aware penalty, and *S_i_*is the adaptive scale factor. The corresponding box regression loss is computed over the foreground predictions as Equation 8.

(8) $${ℒ}_{\text{box}}=\frac{\sum_{i\in ℱ}u_{i}\left(1-I_{AGS,i}\right)}{\sum_{i\in ℱ}u_{i}+\epsilon },$$

where *u_i_*is the assignment weight of the *i*-th foreground prediction. The total detection objective is Equation 9.

(9) $${ℒ}_{\text{det}}=g_{\text{box}}{ℒ}_{\text{box}}+g_{\text{cls}}{ℒ}_{\text{cls}}+g_{\text{DFL}}{ℒ}_{\text{DFL}},$$

where ${ℒ}_{\text{cls}}$ is the binary classification loss, ${ℒ}_{\text{DFL}}$ is the distribution focal loss for box-distance distribution learning (Li et al., 2020), and *g*_box_, *g*_cls_, and *g*_DFL_ are the loss gains. By modifying only ${ℒ}_{\text{box}}$, AGS-IoU supplies direction-, shape-, and quality-aware supervision for bounding-box regression while preserving the lightweight inference pathway of RPB-YOLO11.

### 2.6 Evaluation metrics

The evaluation protocol was designed to measure both detection reliability and deployment-oriented model complexity. Because the task contains two object categories, C={spike_blast,health}, all detection metrics were computed over class-wise detections and then averaged across classes. A predicted box was considered for class *c* at IoU threshold *τ*. It was counted as a true positive only when its class label matched an unmatched ground-truth box and its IoU with that box was not smaller than *τ*. Remaining detections were counted as false positives, and unmatched ground-truth boxes were counted as false negatives. This protocol follows the standard object-detection evaluation logic used in the PASCAL VOC and COCO benchmarks (Everingham et al., 2010; Lin et al., 2014).

For a class *c*, IoU threshold *τ*, and confidence operating point *ρ*, three counts were used after retaining predictions with confidence at least *ρ*. These counts were ${\text{TP}}_{c,\tau }\left(\rho \right)$, ${\text{FP}}_{c,\tau }\left(\rho \right)$, and. ${\text{FN}}_{c,\tau }\left(\rho \right)$ Precision and recall were defined as Equation 10.

(10) $$\begin{array}{ll} P_{c,\tau }\left(\rho \right) & =\frac{TP_{c,\tau }\left(\rho \right)}{TP_{c,\tau }\left(\rho \right)+FP_{c,\tau }\left(\rho \right)+\epsilon },\\ R_{c,\tau }\left(\rho \right) & =\frac{TP_{c,\tau }\left(\rho \right)}{TP_{c,\tau }\left(\rho \right)+FN_{c,\tau }\left(\rho \right)+\epsilon }, \end{array}$$

where *P_c,τ_* measures the reliability of retained detections and *R_c,τ_* measures the fraction of annotated objects recovered by the detector. The reported precision and recall values were class-averaged at *τ* = 0.50, which prevents the more frequent health class from alone determining the summary metric. *ϵ* is a small constant used to avoid division by zero.

Average precision was calculated from the precision-recall curve obtained by sweeping the confidence threshold. For each class and IoU threshold, the precision envelope was integrated over recall Equation 11:

(11) $${\text{AP}}_{c,\tau }=\int_{0}^{1}P_{c,\tau }^{\text{env}}\left(r\right)\;dr,$$

where $P_{c,\tau }^{\text{env}}\left(r\right)$ is the interpolated precision envelope at recall level *r*. In practice, AP was estimated with a 101-point interpolation over *r* ∈ [0,1], consistent with the COCO-style detection metric (Lin et al., 2014). The main overlap metric used in the ablation and comparison tables was mAP50, defined as mean average precision at IoU 0.50 Equation 12:

(12) $$\text{mAP}50=\frac{1}{\left|C\right|}\sum_{c\in C}{\text{AP}}_{c,0.50}.$$

To provide a stricter localization measure, mAP50–95 was also computed as mean average precision over ten IoU thresholds from 0.50 to 0.95 Equation 13:

(13) $$\text{mAP}50-95=\frac{1}{10\left|C\right|}\sum_{\tau \in T}\sum_{c\in C}{\text{AP}}_{c,\tau },\;T=\left\{0.50,0.55,\ldots ,0.95\right\}.$$

Equation 12 emphasizes whether panicle instances are localized with the conventional 0.50 IoU criterion. Equation 13 evaluates localization quality under progressively stricter overlap requirements.

Model compactness was evaluated with parameter count and GFLOPs. Parameter count denotes the number of trainable weights and reflects model storage and memory pressure. GFLOPs were measured at the 640-pixel input setting and approximate arithmetic cost under a fixed image size. These two indicators were considered together with precision, recall, mAP50, and mAP50–95 so that improvements in rice panicle blast detection could be interpreted against the computational constraints of edge deployment (Howard et al., 2017; Tan et al., 2020).

### 2.7 Android platform deployment

The Android platform was designed to provide local image inference for rice panicle blast survey without requiring remote server communication. This deployment setting is consistent with edge-intelligence applications, where model execution is moved closer to the sensing device to reduce data transmission and support field use under limited connectivity (Shi et al., 2016; Zhou et al., 2019; Merenda et al., 2020). The target workflow was image acquisition, on-device preprocessing, RPB-YOLO11 inference, detection post-processing, visual overlay, and panicle-level statistical reporting. This route extends the mobile plant disease diagnosis concept from image-level judgement to object-level localization and counting (Johannes et al., 2017).

The trained RPB-YOLO11 model was exported for mobile inference through a TorchScript route and embedded as an application asset. ONNX export was also generated and checked for consistency, but the Android implementation evaluated in this study used the TorchScript model. The mobile input pipeline resized each selected or captured image to 640 × 640 pixels, converted the image to RGB channels, and normalized pixel values to the range [0,1]. The prediction tensor was interpreted as bounding-box coordinates and class confidence scores for the two categories defined in Section 2.1. Candidate detections were filtered by confidence, refined with class-wise non-maximum suppression, and mapped back to the original image coordinate system before visualization.

The application output was organized for field phenotyping rather than detector inspection alone. For each image, the platform displayed bounding boxes, class labels, confidence scores, and class counts for spike_blast and health. It also summarized the disease incidence among detected panicles. Let *N*_d_ denote the number of detected diseased panicles and *N*_t_ denote the total number of detected panicles. When *N*_t_ *>* 0, the image-level IDP was calculated as Equation 14.

(14) $$\text{IDP}=\frac{N_{d}}{N_{t}}\times 100\%.$$

When no panicle was detected, the platform did not assign a disease-incidence conclusion. Otherwise, the IDP value was mapped to the rice blast resistance-grade thresholds specified in the agricultural industry standard NY/T 2646-2014, *Technical specification for identification and evaluation of blast resistance in rice variety regional test* (Ministry of Agriculture of the People’s Republic of China, 2014). The standard threshold table used by the application is shown in **Table 1**. The implementation followed these thresholds directly. It did not apply an additional tolerance interval, so the HR grade was assigned only when IDP = 0%.

**Table 1:** Rice panicle blast resistance-grade thresholds based on the diseased-panicle incidence standard in NY/T 2646-2014.

Grade	Resistance or susceptibility type	Diseased-panicle incidence (%)
0	Highly resistant (HR)	0
1	Resistant (R)	≤ 5.0
3	Moderately resistant (MR)	5.1–10.0
5	Moderately susceptible (MS)	10.1–25.0
7	Susceptible (S)	25.1–50.0
9	Highly susceptible (HS)	≥ 50.1

A complete phenotyping validation would require paired manual references for the same images, quadrats, or plots. For each sampling unit, trained observers should record manual diseased-panicle counts, totalpanicle counts, or standard resistance grades. The IDP calculated from the application should then be compared with the manual reference by reporting mean absolute error, root mean squared error, correlation, and Bland–Altman mean bias with limits of agreement. These statistics are necessary because objectdetection accuracy alone does not prove that the downstream incidence index agrees with field-survey practice.

The Android evaluation used an Android API 35 emulator with the packaged TorchScript application. The application started, loaded the bundled model, accepted gallery- or camera-style image input, performed local inference, displayed detection overlays, reported spike_blast/health/total counts, and saved the displayed result image. Latency on physical phones was also evaluated on a Xiaomi 14 and an OPPO Reno13 using five test images. Full-process latency per image included image loading, inference, and visualization.

## 3 Results and analysis

### 3.1 Comparison of detection performance across different models

RPB-YOLO11 was compared with the original YOLO11n baseline, representative lightweight YOLO models, and RT-DETRv2-S on the test set. The YOLO models followed the same training protocol. RTDETRv2-S was included as a larger reference detector. The comparison used precision, recall, mAP50, mAP50-95, parameter count, and GFLOPs as the main indicators. These metrics measure detection accuracy and the computational scale needed for edge deployment. **Table 2** summarizes the comparison results.

**Table 2:** Comparison of detection performance across different models on the test set.

Model	Precision(%)	Recall(%)	mAP50(%)	mAP50-95(%)	Params (M)	GFLOPs
*YOLO-series lightweight detectors*						
YOLO11n baseline	72.34	70.64	73.36	43.32	2.583	6.31
YOLOv5n	69.80	68.89	72.10	41.04	2.182	**5.80**
YOLOv6n	**75.74**	67.41	72.50	43.04	4.155	11.47
YOLOv8n	74.07	67.15	71.58	41.00	2.685	6.82
YOLOv9t	73.30	65.90	71.76	41.58	**1.730**	6.44
YOLOv10n	72.50	60.91	67.96	40.20	2.695	8.23
RPB-YOLO11	73.98	**72.75**	**76.09**	**45.44**	2.875	6.21
*Transformer-based reference detector*						
RT-DETRv2-S	**78.80**	**73.00**	**77.40**	**49.70**	20.08	61.16

Precision, recall, mAP50, and mAP50–95 are reported as percentages. Params denotes trainable parameters in millions. GFLOPs denotes computational cost at the tested input setting. Bold values in the YOLO-series block indicate the best result within that block. RT-DETRv2-S is listed separately as a larger transformer-based reference detector. Bold values indicate that the model performs best on this metric.

Compared with the YOLO11n baseline, RPB-YOLO11 increased precision from 72.34% to 73.98%, recall from 70.64% to 72.75%, mAP50 from 73.36% to 76.09%, and mAP50–95 from 43.32% to 45.44%. These changes corresponded to gains of 1.64, 2.11, 2.73, and 2.12 percentage points, respectively. The improvement involved both fewer missed panicle instances and higher average localization accuracy. For field phenotyping, missed diseased or healthy panicles can directly affect downstream disease-incidence estimates.

In the full comparison, RT-DETRv2-S achieved the highest detection accuracy. It reached 78.80% precision, 73.00% recall, 77.40% mAP50, and 49.70% mAP50-95. Among the YOLO models, RPBYOLO11 achieved the highest recall, mAP50, and mAP50-95. YOLOv6n and YOLOv8n had higher precision than RPB-YOLO11, but both had lower recall and average precision. YOLOv9t had the smallest parameter count, and YOLOv5n had the lowest GFLOPs.

The YOLOv6n result showed that a larger lightweight detector was not automatically better for this dataset. Its relatively high precision was accompanied by lower recall and lower mAP50. This pattern indicates more conservative detection behavior on the test set. It may have missed more panicle instances in scenes with sparse, elongated, and partly occluded targets. This pattern is task-specific and does not imply a general ranking of model architectures.

Model complexity changed the interpretation of the accuracy results. RT-DETRv2-S used 20.08 M parameters and 61.16 GFLOPs. RPB-YOLO11 used 2.875 M parameters and 6.21 GFLOPs. The larger detector used about 7.0 times more parameters and 9.8 times more computation. The accuracy gap may also relate to the detector architecture. RT-DETRv2-S can use transformer-style global context and query matching to refine boxes in crowded scenes. This helps in images with overlapping panicles or leaf-like backgrounds, but it also increases computation. RPB-YOLO11 stays compact and efficient, so it is better suited to mobile field survey.

**Figure 6:**
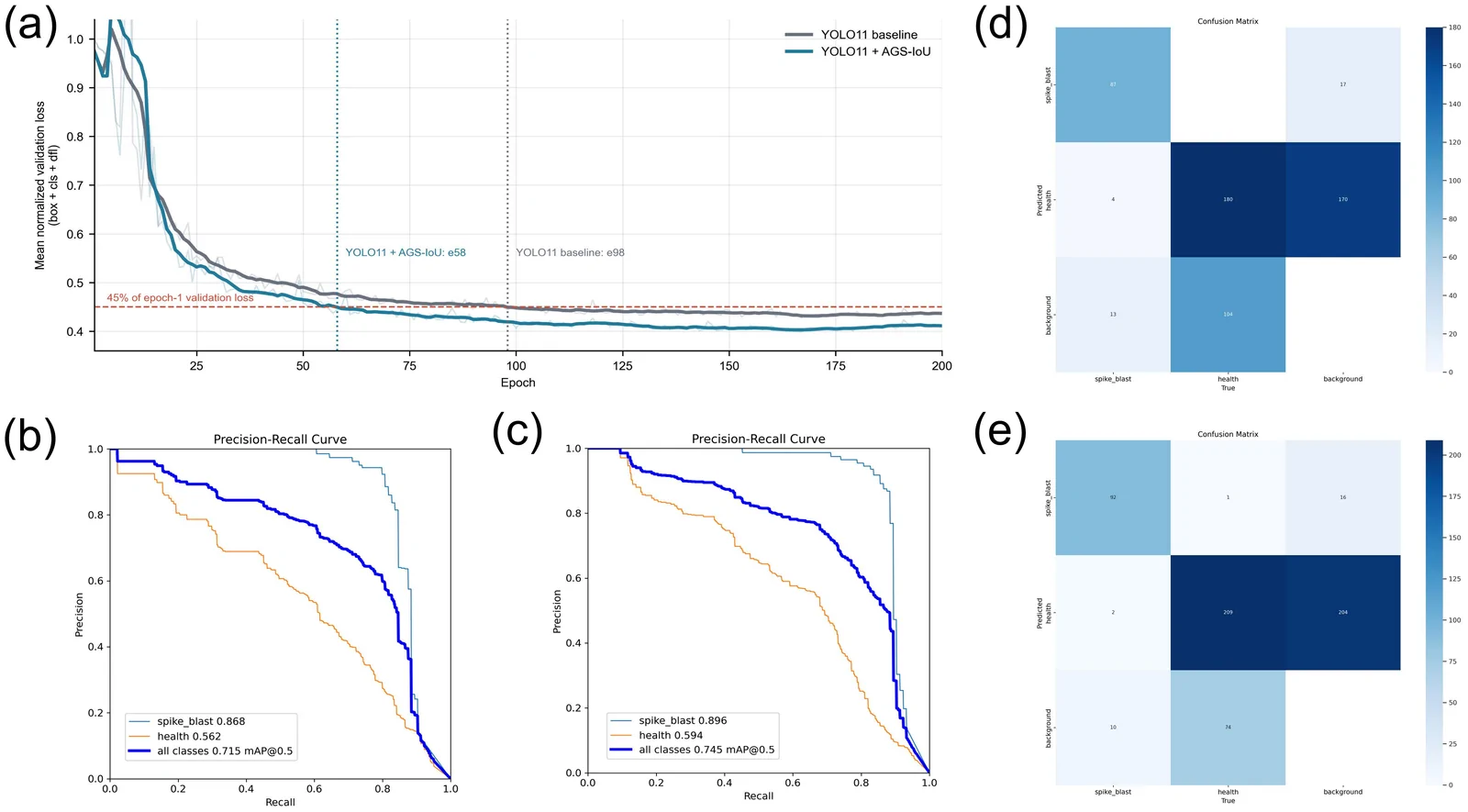
Curve-level and confusion-matrix diagnostics between the YOLO11n baseline and RPBYOLO11. **(a)** Mean normalized validation loss curves. **(b, c)** Validation-set precision-recall curves of the baseline and RPB-YOLO11, respectively. **(d, e)** Validation-set confusion matrices of the baseline and RPB-YOLO11, respectively.

**Figure 7:**
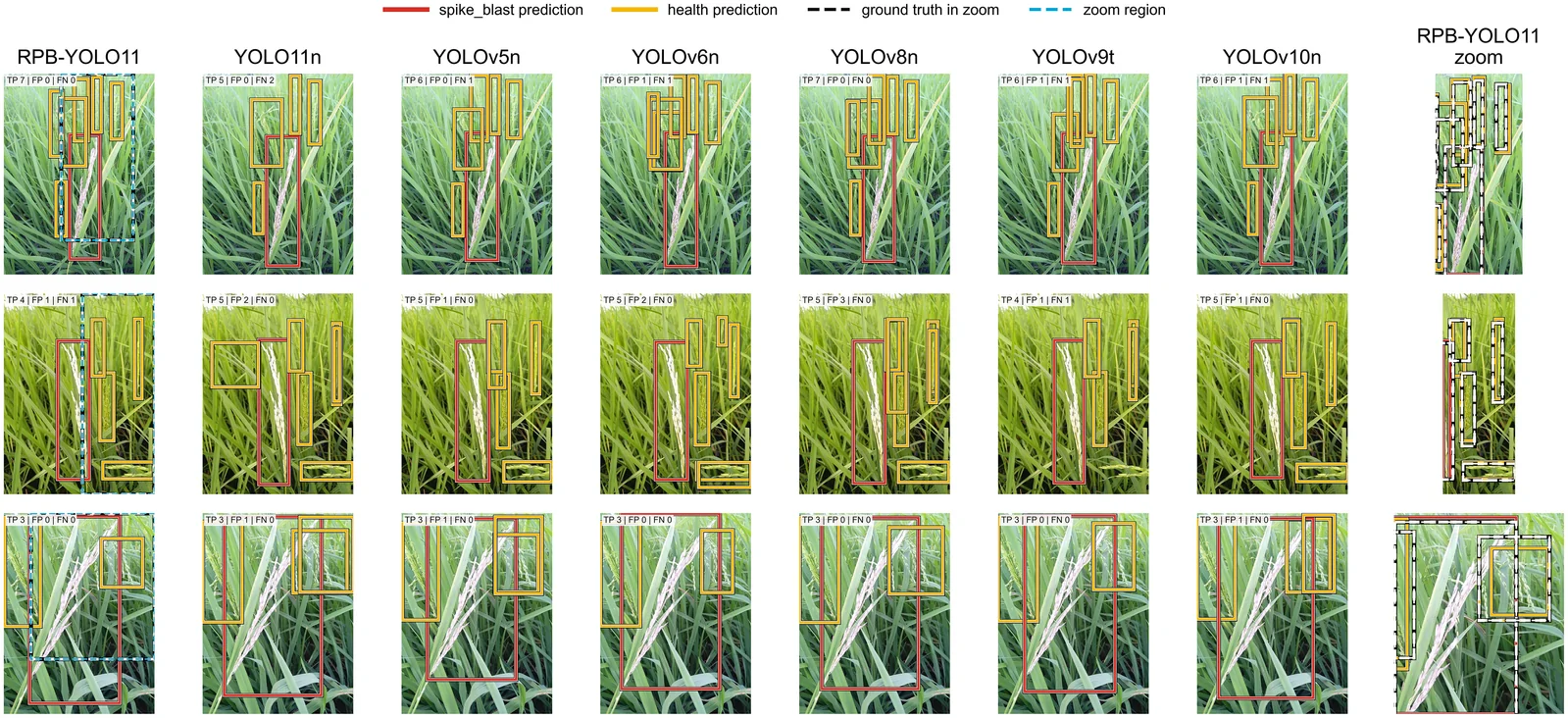
Qualitative detection comparison on representative test images. The same image examples are shown for RPB-YOLO11 and six YOLO comparison models. Red boxes indicate spikeblast predictions, and yellow boxes indicate health predictions. Black dashed boxes mark ground-truth boxes in the zoomed panels, and cyan dashed rectangles mark the zoomed regions.

**Figure 8:**
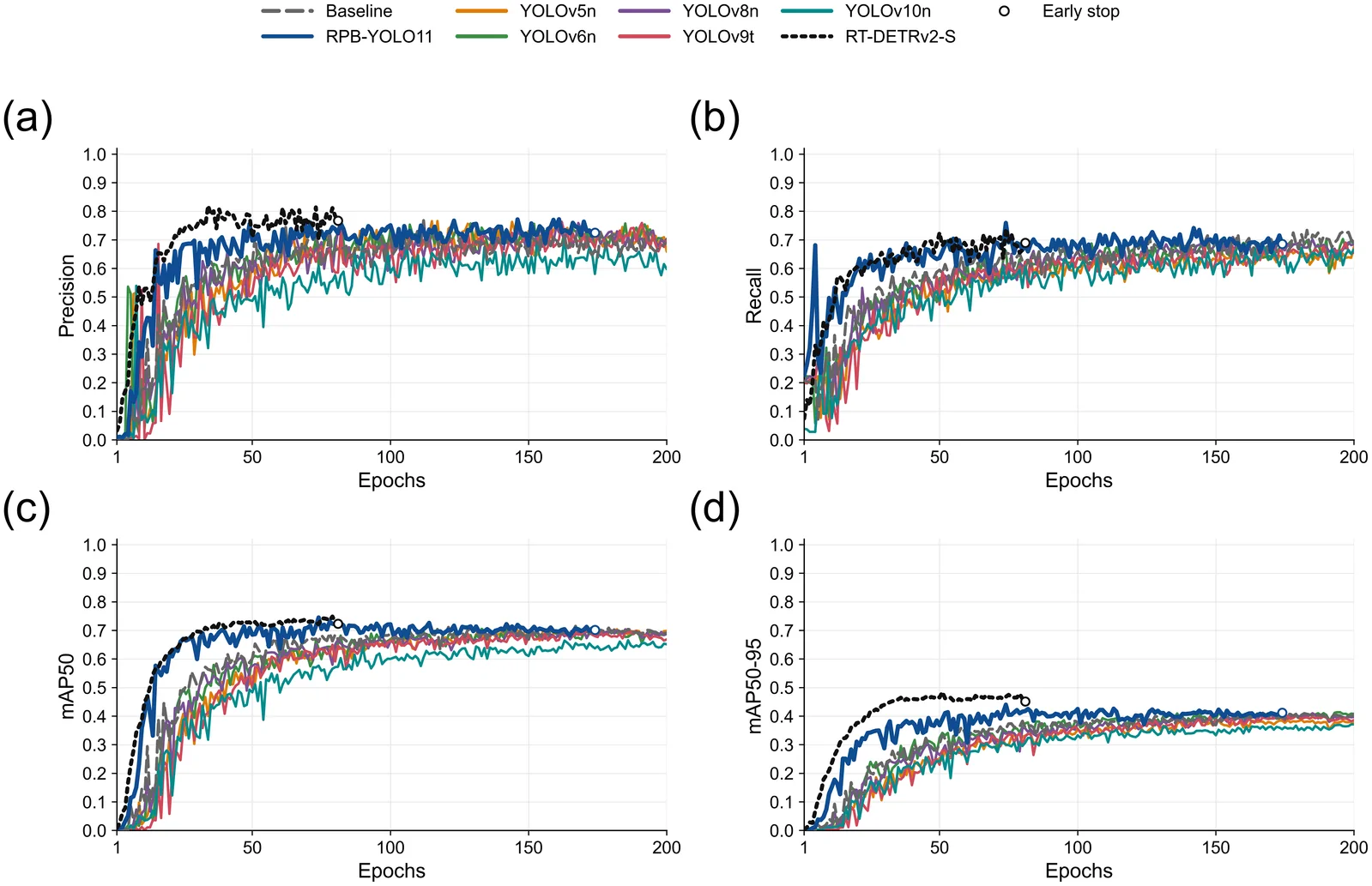
Training metric curves of RPB-YOLO11, the YOLO comparison models, and RT-DETRv2-S. **(a)** Precision, **(b)** recall, **(c)** mAP50, and **(d)** mAP50-95. Curves are shown over the epochs completed by each model. Open circles mark early-stopped endpoints.

**Figure 9:**
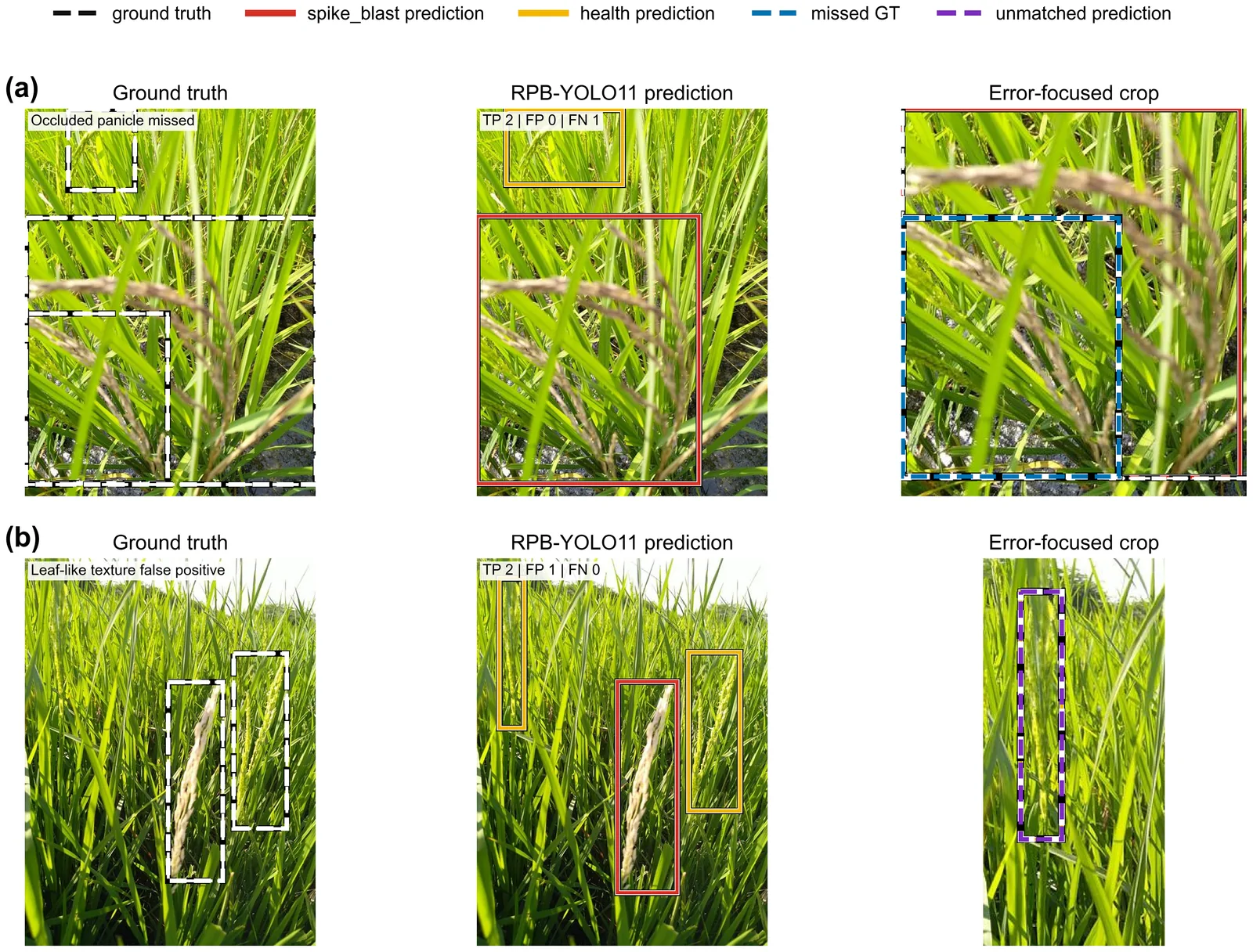
Representative failure cases of RPB-YOLO11 on the test set. **(a)** A missed detection under strong panicle occlusion. **(b)** An unmatched prediction caused by leaf-like texture. Ground-truth boxes are shown with black dashed outlines, spikeblast predictions in red, health predictions in yellow, missed ground truth in blue dashed outlines, and unmatched predictions in purple dashed outlines. Errors were assigned by one-to-one matching at IoU 0.50.

**Figures 6**–**9** summarize the diagnostic, qualitative, training-curve, and failure-case evidence. The qualitative comparison uses the same image examples across all YOLO models. Yellow health boxes and RPB-YOLO11 zoomed regions make small panicles easier to distinguish from background leaves. In these examples, RPB-YOLO11 gave complete localization in the first and third images, but still retained one missed target and one unmatched prediction in the second image. In the training curves, RPB-YOLO11 kept stronger recall, mAP50, and mAP50–95 than the other YOLO models. RT-DETRv2-S rose quickly during training and showed higher AP values.

The failure cases in **Figure 9** define the remaining boundary conditions of the detector. Severe occlusion can hide part of an elongated panicle and reduce the visible evidence needed for matching. Leaf-like textures can also produce an elongated response similar to a healthy panicle. These cases help explain why the gain in mAP50–95 was smaller than the gain in mAP50. Under stricter IoU thresholds, small boundary shifts on narrow panicle boxes are penalized more strongly. Module-level contributions are examined in the ablation analysis below.

Overall, the comparison showed two main patterns. RT-DETRv2-S gave the best absolute accuracy, consistent with broader context modeling and query matching, but it required much higher computation. RPB-YOLO11 gave the best recall and AP values among YOLO models while staying close to the baseline cost. This balance matches the target use case of field-image phenotyping, where the detector should recover panicle instances without excessive computation. This comparison reflects full-detector behavior, and component-level effects are separated in the ablation experiments below.

### 3.2 Ablation study

To clarify how the proposed components contributed to rice panicle blast detection, an ablation study was conducted by progressively enabling the lightweight backbone package, FMSA, and AGS-IoU. In the ablation design, the LGB/A2SA column represents the improved backbone package, where the lightweight Ghost-based backbone and A2SA high-level attention are used together. This grouping is reasonable because A2SA is inserted after the backbone output and was designed to compensate for the directional and high-level representation demands introduced by rice panicle morphology. **Table 3** reports the ablation results.

**Table 3:** Ablation study of the main components in RPB-YOLO11. The LGB/A2SA column denotes the improved lightweight backbone package that includes LGB and A2SA.

Model	LGB/A2SA	FMSA	AGS-IoU	Precision(%)	Recall(%)	mAP50(%)	mAP50-95(%)	Params (M)	GFLOPs
YOLO11 baseline				72.34	70.64	73.36	43.32	2.583	6.31
YOLO11 + LGB + A2SA	✓			75.06	67.47	74.44	42.82	2.860	**6.18**
YOLO11 + FMSA		✓		71.93	70.49	72.37	43.82	2.598	6.35
YOLO11 + AGS-IoU			✓	71.99	68.92	72.62	43.78	2.583	6.31
YOLO11 + LGB + A2SA + FMSA	✓	✓		**78.05**	67.64	74.22	42.02	2.875	6.21
YOLO11 + LGB + A2SA + AGS-IoU	✓		✓	75.72	71.43	75.17	45.10	2.860	**6.18**
YOLO11 + FMSA + AGS-IoU		✓	✓	71.52	68.96	72.00	44.11	2.598	6.35
RPB-YOLO11	✓	✓	✓	73.98	**72.75**	**76.09**	**45.44**	2.875	6.21

Precision, recall, mAP50, and mAP50–95 are reported as percentages. Params denotes trainable parameter count in millions, and GFLOPs denotes computational cost.The two-component combinations revealed how the modules interacted. LGB/A2SA combined with FMSA produced the highest precision in the ablation study (78.05%), but recall remained 67.64% and mAP50–95 decreased to 42.02%. LGB/A2SA combined with AGS-IoU was the strongest two-component variant, reaching 75.17% mAP50, 45.10% mAP50-95, and 71.43% recall with 6.18 GFLOPs. FMSA combined with AGS-IoU increased mAP50–95 to 44.11%, but its mAP50 and recall were lower than the baseline. These patterns show that module interactions affected the balance between confident detections, object recovery, and strict localization. Bold values indicate that the model performs best on this metric.

The single-component experiments showed distinct trade-offs rather than uniform improvements across all metrics. The LGB/A2SA backbone package increased precision from 72.34% to 75.06% and mAP50 from 73.36% to 74.44%, while reducing GFLOPs from 6.31 to 6.18. Recall decreased to 67.47%, and mAP50–95 decreased slightly to 42.82%. This result indicates that lightweight feature extraction and directional high-level refinement improved confident detections and arithmetic efficiency, but they were not sufficient alone to recover difficult panicle instances.

FMSA and AGS-IoU affected stricter localization differently. When FMSA was used alone, mAP50–95 increased from 43.32% to 43.82%, while precision, recall, and mAP50 remained close to or below the baseline. AGS-IoU alone showed a similar pattern, increasing mAP50–95 to 43.78% without changing inference GFLOPs, but reducing precision, recall, and mAP50. These results indicate that either scalesensitive neck refinement or geometry-aware regression can improve localization quality at higher IoU thresholds, but neither single modification was sufficient to improve the full metric set.

The full RPB-YOLO11 model produced the best overall result, with 72.75% recall, 76.09% mAP50, and 45.44% mAP50–95 while maintaining 6.21 GFLOPs. Compared with the baseline, precision increased by 1.64 percentage points, recall by 2.11 percentage points, mAP50 by 2.73 percentage points, and mAP50–95 by 2.12 percentage points. The full model did not maximize precision or minimize GFLOPs; instead, it achieved the strongest recovery and average-precision performance among the ablation variants. Rice panicle blast survey depends on both diseased and healthy panicle counts, so this balance is more useful for field phenotyping than optimizing precision alone.

The ablation study showed that the three modifications played different roles. LGB/A2SA mainly improved precision, mAP50, and computational efficiency. FMSA contributed to stricter localization when used alone. AGS-IoU improved mAP50–95 without adding inference operators. The highest recall, mAP50, and mAP50–95 were achieved only when the three components were used together. Their combination was more effective than optimizing any single part of the detector. The current analysis is still based on overall model metrics. Further evaluation under different object sizes, occlusion levels, and disease severities is needed to better understand the contribution of each component in field conditions.

### 3.3 RPB-YOLO11-based Android field survey tool

**Figure 10:**
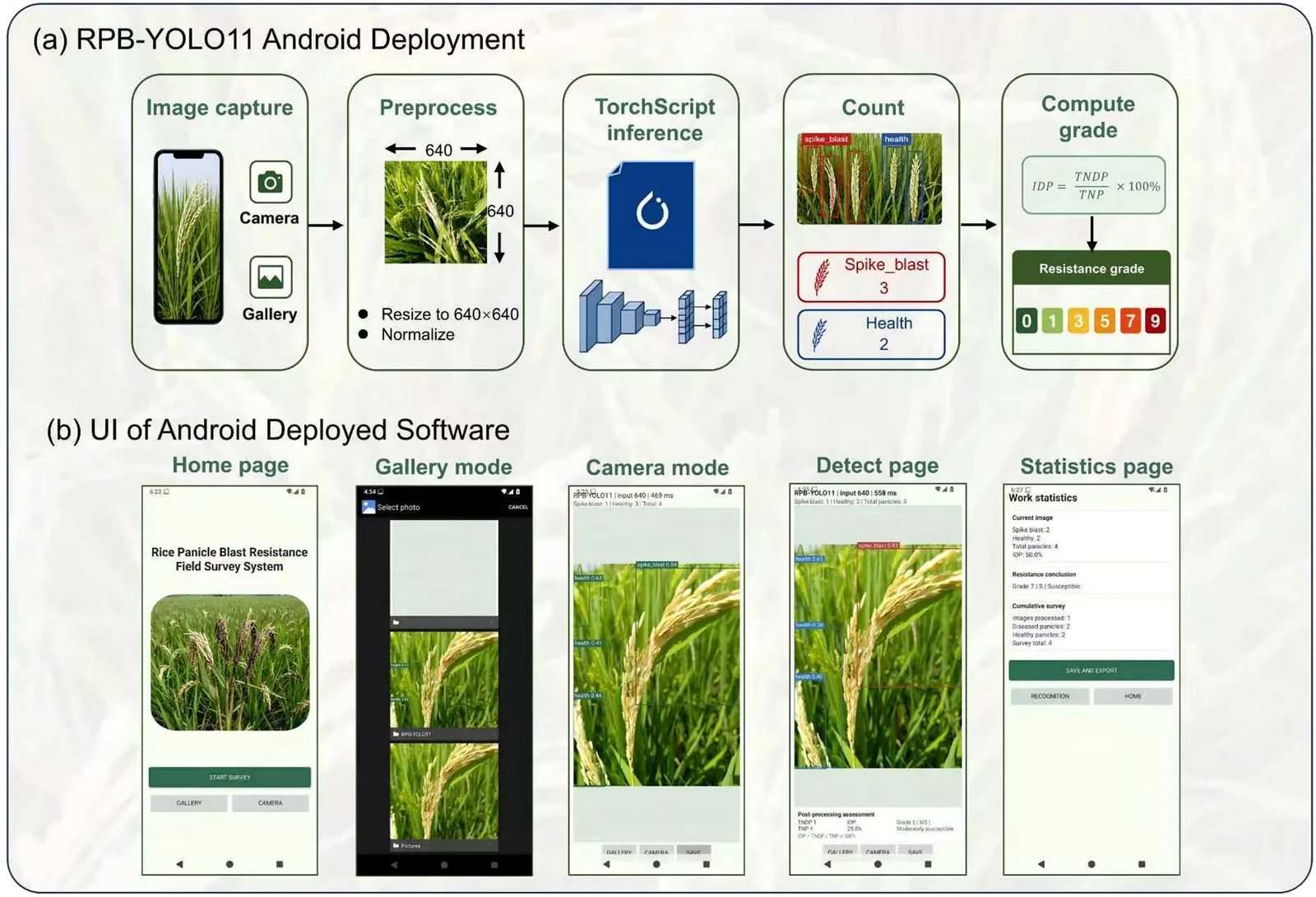
Android deployment route and user interface of the RPB-YOLO11-based field survey tool. **(a)** Local workflow from image capture and preprocessing to mobile inference, panicle counting, IDP calculation, and resistance-grade output. **(b)** Representative interface states, including the home page, gallery mode, camera mode, detection page, and statistics page.

In the emulator, the application executed the complete workflow described in Section 2.7 and **Figure 10**. It loaded the packaged detector, accepted image input, generated detection overlays, displayed confidencelabeled boxes, reported category-wise counts, and saved the resulting image. In the held-out example, the tool detected one diseased panicle and three healthy panicles, giving a total of four panicles. According to Eq. (14), this output corresponded to an image-level IDP of 25.0%, which falls within the moderately susceptible threshold in **Table 1**. The displayed inference latency ranged from 469 to 518 ms per image in the emulator environment (**Table 4**). On physical phones, the full process latency was 1.17 ± 0.32 s per image on the Xiaomi 14 and 1.51 ± 0.70 s per image on the OPPO Reno13. These values were measured on five test images per phone and included image loading, inference, and visualization (**Table 5**). These observations show that detector output can be transformed into quantitative survey information on an Android platform.

**Table 4:** Deployment validation and survey-output parameters of the RPB-YOLO11-based Android field survey tool.

Item	Setting or observed result
Validation environment	Android API 35 x86_64 emulator
Mobile inference route	PyTorch Android 2.1.0 with the packaged RPB-YOLO11 mobile model
Input specification	RGB image resized to 640 × 640 pixels and normalized to [0,1]
Detection categories	Spike_blast and health
Prediction representation	YOLO prediction tensor of size 1 × 6 × 8400
Filtering configuration	Confidence threshold of 0.25 and category-wise non-maximum suppression threshold of 0.45
Survey outputs	Bounding boxes, category labels, confidence scores, diseased-panicle count, healthy-panicle count, total panicle count, IDP, resistance grade, and saved visual result
Incidence result for the example image	IDP = 25.0%, calculated using Eq. (14)
Latency in the emulator	469–518 ms per image

The values summarize the Android workflow evaluated in the emulator environment.

**Table 5:** Physical-phone latency benchmark of the RPB-YOLO11-based Android field survey tool. Latency is reported as mean ± standard deviation.

Device	Processor	Images	Measurement scope	Latency
Xiaomi 14	Snapdragon 8 Gen 3	5	Image loading, inference, and visualization	1.17 ± 0.32 s/image
OPPO Reno13	MediaTek Dimensity 8350	5	Image loading, inference, and visualization	1.51 ± 0.70 s/image

The value of the tool lies in connecting object detection with panicle-level disease phenotyping. A binary image-level diagnosis can indicate whether symptoms are present, but it cannot provide the denominator required for estimating diseased-panicle incidence. By detecting both spikeblast and health instances, the Android workflow produced *N*_d_, *N*_t_, and IDP in the same inference cycle. The visual overlay also preserved spatial evidence for each counted object, which supports later checking of missed detections, false positives, and ambiguous panicle boundaries. This traceability is particularly relevant in dense paddy scenes, where overlapping panicles and similar background textures can otherwise make disease-count records difficult to audit.

From a deployment perspective, the Android validation supports the edge-phenotyping motivation of RPB-YOLO11. Local inference reduces dependence on network transmission and is consistent with edgeintelligence systems designed to move computation closer to the sensing device (Shi et al., 2016; Zhou et al., 2019; Merenda et al., 2020). The low GFLOPs reported in Sections 3.1 and 3.2 support physical-device deployment, and the phone benchmark provides initial runtime evidence on two consumer devices. The Android tool links detection accuracy with field-facing survey output, while broader device testing remains necessary.

The Android implementation demonstrated system integration and survey-output generation, but it remains an initial deployment evaluation rather than a full field trial. The phone benchmark covered two devices and five test images per device. Broader operational claims require further validation under real paddy-field imaging conditions.

## 4 Conclusion

This study proposed RPB-YOLO11, a lightweight detector for rice panicle blast detection in field images. The model was designed to improve the detection of diseased spike regions while keeping the computational cost suitable for mobile edge use. In matched experiments on the test set, RPB-YOLO11 achieved 73.98% precision, 72.75% recall, 76.09% mAP50, and 45.44% mAP50–95 with 6.21 GFLOPs. It outperformed the YOLO11n baseline across these detection metrics. RT-DETRv2-S reached higher absolute accuracy, which may reflect its transformer-style matching and broader scene context. RPB-YOLO11 kept the cost much lower, which makes it more practical for mobile field surveys.

These results indicate that RPB-YOLO11 can provide a compact solution for rice panicle blast phenotyping. This setting is sensitive to missed diseased or healthy panicles, because missed instances may affect incidence estimation. The Android implementation further shows that the detector can be connected to a simple image-based survey tool for local inference, result visualization, and basic field recording.

The study remains limited by dataset scale and broader field validation. Severe occlusion and leaf-like textures remain difficult. More diverse images from different sites, cultivars, growth stages, imaging conditions, and disease severities are still needed to evaluate generalization more fully.

## Data Availability

The datasets presented in this study can be found in online repositories. The names of the repository/repositories and accession number(s) can be found below: https://github.com/XuzheYang2Doc/RPB-YOLO11/releases/tag/rpb-yolo11.
